# On the Subjective Acceptance during Cardiovascular Magnetic Resonance Imaging at 7.0 Tesla

**DOI:** 10.1371/journal.pone.0117095

**Published:** 2015-01-26

**Authors:** Sabrina Klix, Antje Els, Katharina Paul, Andreas Graessl, Celal Oezerdem, Oliver Weinberger, Lukas Winter, Christof Thalhammer, Till Huelnhagen, Jan Rieger, Heidrun Mehling, Jeanette Schulz-Menger, Thoralf Niendorf

**Affiliations:** 1 Berlin Ultrahigh Field Facility (B.U.F.F.), Max-Delbrueck-Center for Molecular Medicine, Berlin, Germany; 2 Experimental and Clinical Research Center (ECRC), a joint cooperation between the Charité Medical Faculty and the Max-Delbrueck-Center for Molecular Medicine, Berlin, Germany; 3 HELIOS Klinikum Berlin-Buch, Dept. of Cardiology and Nephrology, 13125 Berlin, Germany; University Hospital Essen, GERMANY

## Abstract

**Purpose:**

This study examines the subjective acceptance during UHF-CMR in a cohort of healthy volunteers who underwent a cardiac MR examination at 7.0T.

**Methods:**

Within a period of two-and-a-half years (January 2012 to June 2014) a total of 165 healthy volunteers (41 female, 124 male) without any known history of cardiac disease underwent UHF-CMR. For the assessment of the subjective acceptance a questionnaire was used to examine the participants experience prior, during and after the UHF-CMR examination. For this purpose, subjects were asked to respond to the questionnaire in an exit interview held immediately after the completion of the UHF-CMR examination under supervision of a study nurse to ensure accurate understanding of the questions. All questions were answered with “yes” or “no” including space for additional comments.

**Results:**

Transient muscular contraction was documented in 12.7% of the questionnaires. Muscular contraction was reported to occur only during periods of scanning with the magnetic field gradients being rapidly switched. Dizziness during the study was reported by 12.7% of the subjects. Taste of metal was reported by 10.1% of the study population. Light flashes were reported by 3.6% of the entire cohort. 13% of the subjects reported side effects/observations which were not explicitly listed in the questionnaire but covered by the question about other side effects. No severe side effects as vomiting or syncope after scanning occurred. No increase in heart rate was observed during the UHF-CMR exam versus the baseline clinical examination.

**Conclusions:**

This study adds to the literature by detailing the subjective acceptance of cardiovascular magnetic resonance imaging examinations at a magnetic field strength of 7.0T. Cardiac MR examinations at 7.0T are well tolerated by healthy subjects. Broader observational and multi-center studies including patient cohorts with cardiac diseases are required to gain further insights into the subjective acceptance of UHF-CMR examinations.

## Introduction

A growing number of reports refer to explorations into cardiovascular magnetic resonance (CMR) at ultrahigh magnetic field strengths (UHF-CMR, B_0_≥7.0T) [1–5]. These developments are fueled by the signal-to-noise ratio advantage inherent to higher magnetic field strengths and supported by enabling RF coil technology in conjunction with novel imaging methodology. Pilot studies and early applications of UHF-CMR include cardiac chamber quantification of the left [6–9] and right ventricle [10]. Other studies demonstrated the feasibility of high spatial resolution coronary artery imaging [11–13], temporally resolved myocardial T_2_* mapping [14,15], parametric imaging of myocardial T_1_ [16,17] and first-pass myocardial perfusion imaging [18]. Explorations into non-proton MR applications involved localized ^31^P cardiac magnetic resonance spectroscopy [19] and cardiac gated sodium imaging of the heart [20,21]. The implications of these pilot studies feed into a broad spectrum of cardiology, radiology, biomedical engineering and other related fields of clinical research. Arguably, it is too early in the development process to make ultimate statements since UHF-CMR is still in its infancy and the potential of UHF-MR is as yet untapped. It is no secret that the advantages of UHF-CMR are sometimes offset by a number of concomitant physics related phenomena and practical obstacles which can make it a challenge to even compete with the capabilities of CMR at lower fields [2,3]. As UHF-CMR applications become increasingly used for research, they should however help to advance the capabilities of MR for the assessment of cardiovascular diseases but still need to continue to be very carefully validated against CMR applications established at 1.5 T and 3.0 T.

*En route* to broader UHF-CMR studies it is of relevance to examine how UHF-CMR examinations are tolerated by subjects. Practical concerns evoked by the physical size, the mere bore length of today’s 7.0 T MR scanner and the paucity of data about ergonomic constraints, (dis)comfort and sensory side effects are driving the notion that UHF-MR constitutes a challenge for subject tolerance of 7.0 T examinations *per se*. Recognizing this potential, UHF-MR institutions observe subjective acceptance during UHF-MR examinations very carefully [22–25]. The lack of data prompted research into human exposure to ultrahigh magnetic fields and related biophysical and biological effects [26–28], vital signs [29,30], cognitive function [30–34], and stress [35]. Explorations into subject tolerance, subjective perception and sensory side effects during UHF-MR examinations include pioneering single- and multi-centre studies covering static magnetic fields of 7.0 T and higher, RF power deposition induced temperature sensations and spatially varying or rapidly switching magnetic field gradients as potential root causes for discomfort [22–25].

While being very important and valuable the results obtained from these pioneering studies are largely constrained to brain imaging [22–25] so that the conclusions drawn on subjective acceptance do not involve the specific characteristics of UHF-CMR. These particularities include the use of local cardiac-optimized transceiver RF coil arrays covering the upper torso by means of anterior and posterior coil sections rather than volume RF coils surrounding the head. This difference in the setup has implications for subjective discomfort and distress potentially caused by space constraints and the weight of the anterior RF coil section, as well as for RF power deposition considerations and for thermal isolation of the upper torso induced by the relatively large-area RF coil covers. It is common in brain MRI to attach a mirror to the head coil, which enhances subject comfort by allowing a view out of the magnet bore or onto a display featuring animations; an approach which is not common in CMR. Unlike brain MRI the head is not positioned in the magnet’s iso-center for UHF-CMR which might affect the propensity to vertigo, dizziness, metallic taste and light flashes. Likewise, the travel distance of the subject being positioned on the patient table from the home position to the target position is pronounced for UHF-CMR versus brain imaging. With the upper torso being positioned in the magnet’s iso-center for an UHF-CMR examination rapidly switching magnetic field gradients bear the potential to provoke peripheral nerve stimulations (PNS) rates owing to induced electric currents in the body which might differ from PNS rates and regions reported for brain UHF-MR. Extra sensors, ancillary hardware and cabling used to record and track physiological motion for gating/triggering might provide another factor that governs subjective tolerance during UHF-CMR.

Realizing the limitations of previous reports on subjective tolerance during UHF-MR and recognizing the particularities of cardiac MR this study examines the subjective acceptance during UHF-CMR examinations. To meet this goal, a cohort of healthy 165 subjects who underwent a cardiac MR examination at 7.0 T in our institution was asked to fill out a questionnaire under supervision of a study nurse.

## Materials and Methods

### Subjects and subject preparation

Within the period January 2012 to June 2014 a total of 165 healthy volunteers without any known history of cardiac disease underwent UHF-CMR. All volunteers underwent a medical informed consent discussion including a basic clinical examination prior to the UHF-CMR session. Medical history was taken by a clinician. The basic clinical examination included the recording and documentation of height, body weight, body mass index, heart rate, blood pressure, temperature, sex and date of birth in a Case Report Form (CRF). Female subjects underwent a pregnancy test. If this test indicated pregnancy subjects were excluded. Contraindications for UHF-MR were observed very carefully. Subjects with cardiac pacemakers, tattoos, conducting implants or metal clips were excluded. Prior to the actual MR investigation subjects were informed about potential side effects covering vertigo, nausea, dizziness, metallic taste, light flashes, peripheral nerve stimulation and feeling of cold or heat. This standardized information was provided by the same study nurse. No mention was made that these sensations might be more pronounced versus clinical 1.5 T or 3.0 T MR scanners. All subjects were advised that the MR images acquired during the UHF-CMR session are not used for diagnostic purposes. To meet data protection requirements, data were rendered pseudoanonymized.

Each subject was asked to wear MR safe clothes without zippers or snaps provided by our institution. During the examination, the heart rate of each subject was monitored using pulse oximetry and a MR compatible stethoscope (EasyACT, MRI.TOOLS GmbH, Berlin, Germany). The latter was used for cardiac gating and triggering [36–39]. To ensure communication with the research staff operating the scanner, each subject was able to communicate via a two way speaker system and was equipped with an emergency squeeze bulb. For acoustic noise protection each subject received earplugs (3M^TM^ earplugs 1100, Neuss, Germany, noise reduction = -37dB) and headphones (Siemens Healthcare, Erlangen, German, noise reduction = -14dB). Each subject was covered with a blanket reaching up to the torso. The subjects’ head was not fixated but placed on a pad which conveniently conforms to the shape of the head.

### Ethics Statement

For the entire cohort, 165 healthy subjects without known history of cardiac diseases (41 female, 124 male, mean age: 36 ± 12 years, mean BMI: 23.6 ± 3.3 kg/m^2^, mean heart rate: 69 ± 12 bpm) were included after due approval by the local ethical committee (registration number DE/CA73/5550/09; Landesamt für Arbeitsschutz, Gesundheitsschutz und technische Sicherheit, Berlin, Germany). Informed written consent was obtained from each volunteer prior to the study.

### MR equipment at 7 T

All cardiac MR experiments were conducted on a 7.0 T whole body MR scanner (Magnetom, Siemens Healthcare, Erlangen, Germany), with a bore size of 60 cm and a magnet length of 337 cm. The scanner was equipped with a gradient system offering a maximum slew rate of 170 mT/m/ms and a maximum gradient strength of 26 mT/m per axis (Siemens Healthcare, Erlangen, Germany) and an 8-kW single channel RF amplifier (Stolberg HF-Technik AG, Stolberg-Vicht, Germany). A synopsis of the MR system characteristics is listed in Table 1.

Subjects were positioned supine and head-first in the magnet. To reduce if not eliminate side effects caused by spatially-varying magnetic fields the 7.0 T system is equipped with a logic that controls the patient table speed profile. Our measurements showed that a table speed of v_poti_ = 25 mm/s is used when moving the table from its home position towards the magnet’s isocenter. This includes regions with pronounced B_0_*(grad(B_0_)) ranging from approximately 60 cm to approximately 200 cm from the magnet’s isocenter. With the subject being positioned head first our measurements revealed that the table speed changes into v_iso_ = 60 mm/s at a head position of approximately 45 cm from the isocenter. This speed is maintained until the head or heart is positioned in the isocenter.

**Table 1:** Synopsis of the characteristics of the 7.0 T whole body MR system used.

**systems characteristics**	
magnet bore	whole-body magnet
magnet length without cover	337 cm
scanner length including cover	400 cm
inner diameter	60 cm
diameter of flared opening	120 cm
length of flared opening	35 cm
	
**patient table**	
max. patient weight	200 kg
max. range	325 cm
patient table speed	(25–60) mm/s
	
**patient comfort**	
effective inner A-P diameter with patient table in the iso-center position	40 cm
in bore ventilation	can be set to 3 different levels
in bore intercom	including loudspeaker, microphone and earphones

For signal excitation and reception local surface transmit/receive RF coil configurations tailored for ^1^H cardiac MR were employed including (see Table 2 for details):

- a four channel transmit/receive loop coil array [40]
- an eight channel transmit/receive loop coil array [41]
- a sixteen channel transmit/receive loop coil array [9,42]
- a modular 32-channel transmit/receive loop coil array [43]
- an eight channel transmit/receive bow tie antenna array [44]
- a sixteen channel transmit/receive bow tie antenna array [45]

**Table 2:** Overview of the transceiver RF coil arrays (^1^H, f = 298 MHz) coils employed.

RF coil design	number of RF coil elements	RF coil size head-feet x left-right (cm2)	RF coil weight (kg)	max local SAR (10g average) @ 1W input power in (W/kg)	subjects under-going an UHF-CMR examina-tion (n = 165)
**four channel transmit/receive loop coil array**	2 anterior and 2 posterior loop elements	34x30 anterior 35x30 posterior	1.8 anterior 1.7 posterior	0.57	3
					
**eight channel transmit/receive loop coil array**	5 anterior and 3 posterior loop elements	21x31 anterior 21x31 posterior	2.1 anterior 1.8 posterior	0.43	1
					
**sixteen channel transmit/receive loop coil array**	8 anterior and 8 posterior loop elements	33x33 anterior 45x34 posterior	2.1 anterior 2.7 posterior	0.36	50
					
**modular 32-channel transmit/receive loop coil array**	16 anterior and 16 posterior loop elements	32x37 anterior 32x37 posterior	1.6 anterior 1.6 posterior	0.9	40
					
**8 channel transmit/receive bow tie antenna array**	4 anterior and 4 posterior bow tie antennas	15x32 anterior 15x32 posterior	2.6 anterior 2.6 posterior	0.6	26
					
**16 channel transmit/receive bow tie antenna array**	8 anterior and 8 posterior bow tie antennas	31x32 anterior 31x32 posterior	5.3 anterior 5.3 posterior	0.34	45
					

Prior to the volunteer study the RF coil configurations underwent safety assessment to confirm compliance with the relevant sections of IEC 60601–2–33:2010 Ed.3 and IEC 60601–1:2005 Ed.3 [46]. This procedure included numerical electromagnetic field (EMF) simulations together with specific absorption rate (SAR) assessment plus risk assessment and risk management procedures. For EMF simulations a finite integration technique of CST Studio Suite 2011 (CST AG, Darmstadt, Germany) and human voxel models Duke (BMI: 23.1) and Ella (BMI: 22) from the Virtual Family [47] were used to calculate the EMF fields and SAR. A single feeding RF power amplifier transmission mode was used together with fixed phase settings specific for each RF coil [9,40–45].

### Cardiac imaging at 7.0 T

The UHF-CMR protocol included the following protocol as a minimum. Slice positioning was carried out following international consensus [48,49] based upon our previous report [7] For this purpose the heart was localized in three orthogonal thoracic slices placed along each main axis of the upper torso using single breath-hold, low spatial resolution 2D gradient echo acquisitions (matrix = 132 x 192, in-plane spatial resolution = (2.5 x 1.9)mm^2^, slice thickness = 8mm, TR = 6.7 ms, TE = 1.35 ms , FOV = 360 mm, bandwidth = 651Hz/pixel ). The long axis of the left ventricle (LV) was dissected twice, and finally a stack of short axis views was obtained. These slices provided the basis for planning standard long axis views (four-chamber, three-chamber and two-chamber view) derived from 2D CINE FLASH imaging.

Based on the four-chamber view, a mid-ventricular short axis view positioned parallel to the mitral valve plane was planned as a minimum for high spatial resolution CINE imaging. Alternatively a stack of mid-ventricular short axes views covering the complete LV in diastole was positioned parallel to the mitral valve plane. Short axis and long axis CINE views were acquired using single breath-hold 2D CINE FLASH imaging. Imaging parameters are summarized in Table 3.

**Table 3:** Overview of the parameters used for 2D CINE FLASH imaging of the heart at 7.0 T.

RF coils	spatial resolution (mm^3^)	matrix size	FOV (mm^2^)	TE (ms)	TR (ms)	receiver bandwidth (Hz/pixel)	acceleration factor
**four channel TX/RX loop coil array**	(1.4x1.4x4)	256x232	360x326	2.7	5.6	444	1–4
							
**eight channel TX/RX loop coil array**	(1.4x1.4x4)	256x232	360x326	2.7	5.6	444	1–4
							
**sixteen channel TX/RX loop coil array**	(1.4x1.4x4)	256x232	360x326	2.8	6.3	444	1–4
							
**modular 32 channel TX/RX loop coil array**	(1.1x1.1x2.5) (1.4x1.4x4) (1.8x1.8x6)	320x264 232x256 160x176	360x326	3.3 2.7 2.4	6.7 5.6 5.1	446 444 441	1–5
							
**8 channel TX/RX bow tie antenna array**	(1.4x1.4x4)	256x256	360x360	2.7	5.6	444	2
							
**16 channel TX/RX bow tie antenna array**	(0.8x0.8x2.5) (1.1x1.1x2.5) (1.4x1.4x4) (1.8x1.8x6)	380x464 260x320 208x256 170x208	360x326	2.2 2.1 1.7 1.7	4.8 4.6 4.2 4.0	445 446 444 431	1–6

Since these examinations were part of our development process the minimum protocol was supplemented by further research sequences on a case-by case basis. These efforts included localized B_0_ shimming for the assessment of field dispersion across the heart, transmission field mapping, myocardial T_2_* mapping, examination of parallel imaging performance, signal-to-noise ratio assessment, noise correlation measurements and fat-water imaging using methodology and parameters described in [9,10,14,15,42,43]. For all subjects the total duration of the UHF-CMR examination was recorded.

### Assessment of subjective acceptance

For the assessment of the subjective acceptance a questionnaire was used to examine the participant’s experience prior, during and after the UHF-CMR examination. The questionnaire is part of the documents included in our ethics approval. The subjects were kindly asked to respond to the questionnaire in an exit interview held immediately after the completion of the UHF-CMR examination under supervision of a study nurse to ensure accurate understanding of the questions. The questionnaire was setup by strictly following the guidelines given by our IRB approval. All questions were answered with “yes” or “no” including extra space for additional comments as shown in Table 4.

**Table 4:** Questionnaire which was completed by all subjects immediately after the completion of the UHF-CMR examination.

#	Question	yes	no	Comments
1	Did you feel dizziness prior to the study?			
2	Did you feel dizziness during the study?			
3	Did you feel dizziness after the study?			
4	Did you see light flashes?			
5	Did you feel heating?			
6	Did you feel cold?			
7	Did you feel unease?			
8	Did you recognize muscular contraction?			
9	Have you perceived a metallic taste?			
10	Have you noticed other side effects?			

Statistical significance in the differences (i) between male and female subjects, (ii) between the coil configurations used and (iii) between 30 subjects covering the low end of the age range and 30 subjects covering the high end of the age range involved in this study was analyzed using IBM SPSS Statistics 22 (IBM, Ehningen, Germany). Pearson’s analysis (Chi-square test) was applied [50]. A p-value p<0.05 was considered to be statistically relevant significant.

## Results

**Figure 1:**
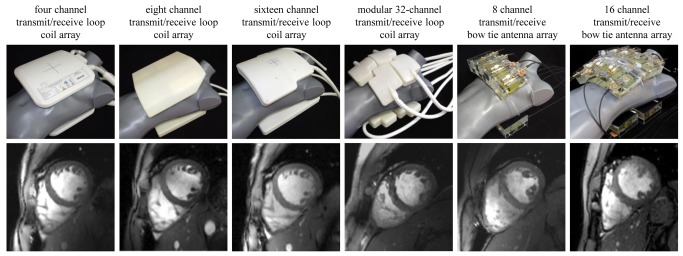
Synopsis of RF coil configurations used in this study. **Top:** Photographs of the cardiac optimized 7.0 T transceiver RF coil arrays to illustrate the coil design and the coil geometry together with the coil positioning used in the UHF-CMR setting. The RF coils employed include a four channel [40], an eight channel [41], a 16 channel [9,42] and a 32 channel loop coil [43] configuration and an eight channel [44] and 16 channel bow tie antenna array configuration [45]. **Bottom:** Short axis views of the heart derived from 2D CINE FLASH acquisitions using the RF coil configurations in the top row and a spatial resolution of (1.4 x 1.4 x 4) mm^3^ and parallel imaging (R = 2, GRAPPA reconstruction).

The subject characteristics are listed in Table 5. During the UHF-CMR examination, a mean heart rate of 64 ± 7 bpm (min: 46 bpm, max: 91 bpm) was observed. In comparison, the clinical examination performed prior to the UHF-CMR exam yielded a mean heart rate of 69 ± 12 bpm (min: 47 bpm, max: 115 bpm). The mean scan time per subject was 64 ± 27min. Examples of short axis views acquired with the 6 RF coil configurations are demonstrated in Fig. 1.

**Table 5:** Overview of subject (n = 165) characteristics.

	total	female	male
**number of questionnaires**	165	41	124
**mean age**	36 ± 12	33 ± 11	37 ± 12
age range min	23	25	23
age range max	72	67	72
**mean height (cm)**	176 ± 9	167 ± 7	179 ± 7
height range min (cm)	158	158	165
height range max (cm)	196	183	196
**mean weight (kg)**	72.9 ± 11.8	63 ± 7.3	76.2 ± 11.1
weight range min (kg)	47.6	47.6	60.5
weight range max (kg)	114.0	83	114.0
**mean BMI (kg/m^2^)**	23.6 ± 3.3	22.7 ± 2.7	23.9 ± 3.5
BMI range min (kg/m^2^)	16.9	18.5	16.9
BMI range max (kg/m^2^)	33.5	32	33.5
**mean blood pressure (mmHg)**	127/79 ± 14/11	123/76 ±14/10	129/80 ± 14/12
blood pressure range min (mmHg)	96/55	96/55	100/55
blood pressure range min (mmHg)	180/119	166/111	180/119
**mean RR-interval (ms) (prior to UHF-MR exam)**	892 ± 149	833 ± 136	912 ± 148
RR-interval range min (ms)	522	618	522
RR-interval range max (ms)	1277	1071	1277
**mean heart rate (bpm) (prior to UHF-MR exam)**	69 ± 12	74 ± 12	68 ± 12
heart rate range min (bpm)	47	56	47
heart rate range max (bpm)	115	97	115
**mean RR-interval (ms) (during UHF-CMR exam)**	945 ± 106	827 ± 78	985 ± 82
RR-interval range min (ms)	657	657	726
RR-interval range max (ms)	1296	1077	1296
**mean heart rate (bpm) (during UHF-CMR exam)**	64 ± 7	73 ± 7	61 ± 5
heart rate range min (bpm)	46	55	46
heart rate range max (bpm)	91	91	82
**mean body temperature (°C)**	36.4 ± 0.5	36.6 ± 0.4	36.3 ± 0.5
temperature range min (°C)	34.5	35.6	34.5
temperature range max (°C)	37.6	37.5	37.6
**mean examination time (min)**	64 ± 27	63 ± 17	65± 27
examination time min (min)	34	45	34
examination time max (min)	124	85	124

No subject aborted the UHF-CMR examination. Throughout the study, there were no injuries or other incidents. A total of 165 questionnaires (male 124, female 41) were completed and included into the analysis.

**Figure 2:**
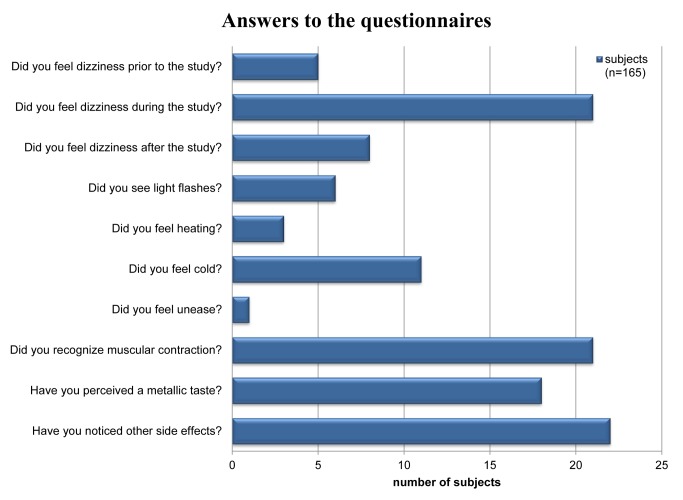
Results derived from the completed questionnaires. Synopsis of the results reported by 165 subjects on subjective acceptance of UHF-CMR. The most mentioned side effects reported were transient muscular contraction during scanning (12.7%) and dizziness experienced during the study (12.7%).

**Figure 3:**
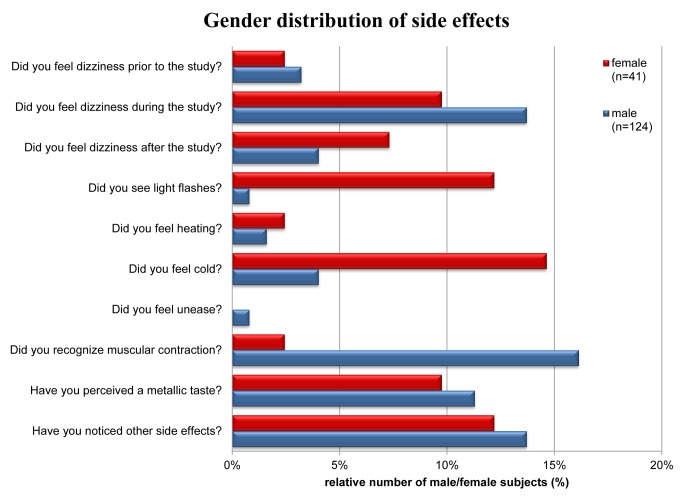
Gender distribution of reasons of discomfort. Synopsis of reasons of discomfort reported by female and male subjects who underwent an UHF-CMR examination.

A synopsis of the evaluation of the questionnaires is provided in Fig. 2. The gender distribution of the reported sensory side effects is shown in detail in Fig. 3. Major differences in response to question 1–9 were not observed for the RF coil configurations used; with the exception of question 8 for which Pearson’s analysis provided p = 0.015.

The analysis of the questionnaires showed that muscular contraction during scanning was found to be among the two most frequently reported side effects. This peripheral nerve stimulation induced sensation was reported by 21 out of 165 subjects which represents 12.7% of the cohort investigated. A closer examination revealed that this phenomenon was reported by one female and 20 male subjects, which corresponds to 2.4% out of all female and 16.1% out of all male subjects. Pearson’s analysis provided p = 0.023 for the gender dependence of muscular contraction. Muscular contraction was reported as a transient symptom. In most cases transient muscular contraction symptoms occurred in the leg, but also in the shoulder or in the abdomen and pelvis. Muscular contraction occurred only during periods of scanning with the magnetic fields gradients being rapidly switched while strictly staying within the specifications and limits for gradient duty cycle, slew rates and maximum gradient amplitudes given by the MR manufacturer.

Dizziness during the study was reported with the same frequency as muscular contraction. A total of 21 subjects (12.7%) documented dizziness experienced during the UHF-CMR examination. This effect was noticed by 4 female (9.8% of female subjects) and 17 male (13.7% of male subjects) subjects. In comparison, 5 subjects (2.4% of female subjects and 3.2% of male subjects) outlined the occurrence of dizziness prior to the UHF-CMR examination. eight subjects (in total 4.8%, 3 female, 5 male) affirmed that dizziness occurred after completion of the UHF-CMR exam. After completion of the study the symptoms were completely resolved within a maximum period of 10 minutes. The subjects which reported dizziness showed an average blood pressure of 135/72 mmHg and an average heart rate of 73 bpm.

Taste of metal was mentioned by 18 subjects which represents 10.1% of the study population. In detail, taste of metal was pointed out by 4/41 female and 14/124 male subjects. Further sensations documented by the subjects in the questionnaires were light flashes which were reported by 6 out of 165 subjects, which correspond to 3.6% of the entire cohort. This includes 5 female (12.2%) and one male (0.8%) subject. Pearson’s analysis provided p = 0.001 for the gender dependence of reports on light flashes.

Feeling of heat was reported by 3 subjects (female: 2.4%, male 1.6%). Feeling of cold was outlined by 11 subjects, which represents 6.6% of the study population. In detail, cold was pointed out by 14.6% of the female and by 4% of the male subjects. Pearson’s analysis provided p = 0.018 for the gender dependence of feeling of cold.

Out of 165 subjects, 22 subjects reported side effects/observations which were not explicitly listed in the questionnaire but covered by the question about other side effects and observations. These comments are summarized in Table 6. We noted that 30% of the subjects belonging to a sub-group of 30 subjects covering the high end of the age range (age 51–72) reported other side effects and observations covered by question 10. In comparison, 6% of the subjects assigned to a sub-group of 30 subjects covering the low end of the age range (age 24–28) reported other side effects and observations covered by question 10. Pearson’s analysis yielded p = 0.034 for the assessment of the age dependence of other side effects and observations covered by question 10.

**Table 6:** Summary of other side effects revealed by the question: “Have you noticed other side effects”.

other side effects (in response to question 10)	total	female	male
arm fell asleep	1	-	1
cough	2	-	2
dry lips	1	-	1
dry mouth	7	1	6
feeling of a caressing on stomach	1	-	1
feet and thighs fell asleep	1	-	1
back pain from lying down with projection into the kidney region	1	1	-
pain in the left ear	1	1	-
seeing shadows	1	-	1
sensory illusions	1	-	1
sensory loss in hip joints	1	-	1
stiff neck	1	1	-
sweating attacks	1	-	1
tingling in both arms	1	1	-
tingling on the forehead	1	-	1
tiredness	1	1	-
vibration at the hip	1	-	1

Seven subjects reported a dry mouth during the UHF-CMR examination. Two subjects reported cough. One subject mentioned that his arm fell asleep during the UHF-CMR examination. One subject was feeling tired and one subject had dry lips. A feeling of a caressing on stomach was mentioned by one male. Feet and thighs fell asleep in one subject. Back pain with projection into the kidney region due to lying on the table was mentioned by one subject. One subject recognized a pain in the left ear, another one saw shadows. Sensory illusion and a sensory loss in the hip joints were reported by one subject in each case. A stiff neck was documented by one subject. One subject reported vegetative symptoms (sweating). Tingling on both arms, and also on the forehead was described once. One subject reported a vibration at the hips. None of the subjects reported claustrophobia. Acoustic noise was not reported as a reason for discomfort. The total UHF-CMR examination time was not reported as a cause for discomfort. None of the subjects reported discomfort induced by the local RF coils placed on the anterior chest.

## Discussion

This study adds to the literature by detailing the subjective acceptance of cardiac magnetic resonance imaging examinations at a magnetic field strength of 7.0 T. Among the two most common experiences that generated discomfort was transient muscular contraction or involuntary muscle twitching due to peripheral nerve stimulation induced by switching of magnetic field gradients. This observation does not accord with previous reports on the subjective tolerance obtained for brain imaging at 7.0 T and at 9.4 T [22,24], which outlined lower incidence rates of transient symptoms of muscular contraction and peripheral nerve stimulation. Notwithstanding this difference these reports included the same gradient coil and technical specifications for maximum slew rate, maximum gradient amplitude and maximum duty cycle used here [22,24]. Even if more powerful gradient coils were used for brain MRI including maximum gradient amplitudes of up to 70 mT/m and slew rates of 400 mT/m/ms twitching and muscular contraction was less frequently reported as a cause of discomfort versus dizziness [24]. It should be noted that the switching frequencies used for CMR are commonly ranging between 300–600 Hz and hence are similar or even below the switching frequencies generally employed for anatomical (approximately 200–600 Hz) and functional brain imaging (approximately 1000–3000 Hz). Also, it is fair to assume that the peak integrals of the magnetic field gradients are smaller for CMR versus brain imaging due to the use of larger slice thicknesses and field of views. However, to meet the speed constraints of CMR it is common that the maximum gradient amplitudes used for slice selection, phase encoding, dephasing read-out and spoiler gradients are larger for CMR versus brain MRI. Our data suggest that male subjects are more strongly affected by involuntary muscle contraction due to peripheral nerve stimulation. This difference might be related to differences in body cross-section which effects the size of the current loops, and hence the magnitude of the current density. An experimental study at a lower magnetic field strength noted that the gender of the subject affects the magnitude of the peripheral nerve stimulation threshold but not the position of the stimulation [51]. Mean stimulation thresholds of healthy male subjects were found to be lower than those of healthy female subjects [51]. This observation was attributed to the larger stature of males [51]. To this end it should be noted, that the average height of the male subjects involved in our study was 7% larger than the average height of the female subjects.

Vertigo was found to be the second effect among the two most frequent causes for discomfort in our UHF-CMR study cohort. Magnetic field related vertigo like sensations are thought to result from magnetic susceptibility differences between vestibular organs and surrounding fluid, and from induced currents acting on the vestibular hair cells [52]. Thresholds for motion induced vertigo have been estimated to be around 1 T/s for greater than 1 s [53]. This translates into a recommendation derived from numerical simulations that a moving speed of 1 m/s should not be exceeded when accessing an area closer than 1 m to the front/rear ends of a 7.0 T magnet [54]. The recent ICNIRP guidelines for limiting exposure to electric fields induced by movement of the human body in a static magnetic field and by time-varying fields below 1Hz recommends that the change of the magnetic flux density B should not exceed 2 T during any 3 s period [55]. For the same reason there is a need to ensure that subjects are moved slowly into the 7.0 T magnet bore resulting in a recommendation that patient table motion is set to be lower than 0.66 T/s. To meet this requirement the speed profile of the table motion is adjusted to B_0_*(grad(B_0_)) by some vendors including the MR system used in our UHF-CMR study. This might explain why previous brain UHF-MR studies reported an incidence rate of approximately 25–34% for vertigo and dizziness [23,56], which is more pronounced than the 12.7% rate observed in our UHF-MR study. In the previous studies subjects were positioned on a non-motorized table and were moved manually into the scanner bore at a constant speed [23,24]. Alternatively, a constant speed was used for automatic table motion [25]. Unlike our UHF-CMR study no attempt was made to reduce the table speed in the vicinity of the highest gradient of the magnetic field [23–25]. The 12.7% incidence rate of vertigo observed in our UHF-MR study is in line with a very recent publication which reported vertigo for 10.5% out of 504 subjects enrolled into UHF-MR examinations [57]. For this study a constant table speed of 20 mm/s [57] was used which is similar to v_poti_ = 25 mm/s used in our study. Our data do not match a very recent report that women are more strongly affected by dizziness in static magnetic fields of 7.0 T [58].

It should be also noted that it is common to use extra pads to reduce bulk head motion in brain UHF-MR. This measure should work in favor of reducing motion induced vertigo. On the other hand, pillows or full size pads that conveniently conform to the shape of the head commonly used in CMR might provide enhanced comfort versus thin layer pads commonly used in brain imaging and hence might contribute to a lower incidence of magnetic field induced vertigo.

In addition to vertigo and nausea being potentially induced by movement in a static magnetic field, a direct interaction of the magnetic field with the vestibular system cannot be excluded [55]. Glover et.al. reported an altered sense of balance for subjects positioned stationary in proximity to a 7.0 T MR magnet [52]. Notwithstanding the potential role of spatially-varying magnetic fields for induction of vertigo, important recent findings provide strong evidence that static magnetic fields stimulate rotational sensors in the brain resulting in involuntary slow-phase eye movements, designated as nystagmus [59] which is thought to share a common mechanism with static magnetic field evoked vertigo [60]. This indicates that magnetic vestibular stimulation makes magnetic field induced nystagmus and vertigo possible while simply lying in the static magnetic field of an MR scanner [59,61]. Nystagmus strength depends on the static magnetic field strength, not motion through the magnetic field. Eye movement measurements using infrared video cameras while the subjects laid still in a 7.0 T MR scanner showed that horizontal nystagmus direction is related to the static head pitch angle, which describes the angulation of the chin towards the chest [59]. For a head pitch angle of approximately 10° to 30°—which resembles a static head pitch commonly used in a CMR setup—a horizontal slow phase eye motion velocity close to zero was observed [59]. In comparison, the largest slow phase eye motion velocity was observed for a head patch angle of approximately -10°; an arrangement which approximately resembles the positioning of the head in a head RF coil [59] used for brain MRI. This phenomenon might provide another plausible interpretation for the decreased occurrence of vertigo reported in our UHF-CMR study versus previous ultrahigh field brain imaging examinations.

Five percent of the 165 volunteers reported that dizziness persisted after the UHF-MR examination but disappeared within 10 min after completion of the study. This observation was underscored by a very recent study, which carefully examined the duration of such effects by quantitatively assessing the vestibular performance including measurements of postural instability and rotational divergences [27]. To this end, a recent report demonstrated that Diphenhydramine—a medication used to prevent motion sickness—reduces the strength of vertigo and nausea in UHF-MR—even at a low dose—and may be even used preventively [62]. Yet, this application should be considered very carefully on a case-by-case basis, taking potential side effects and interactions into account.

Approximately 10% of the study population reported metallic taste which accords very well with previous studies [23]. A similar accordance was found for the occurrences of electromagnetically-induced visual flashes of light due to retina stimulation reported in our study with previous UHF-MR brain imaging studies [24]. None of the subjects reported claustrophobia, which is in agreement with recent UHF-MR studies that reported seven scan abortions due to claustrophobia out of 3467 examinations [24]. This rate might appear to be rather low when compared to previous observations at clinical field strengths of 1.5 T and 3.0 T but can be explained by the solely voluntarily nature of the subject recruitment and the careful preparation of the volunteers.

Acoustic noise was not reported as a reason for discomfort in our study. This observation seems to contradict recent results derived from brain imaging at ultrahigh fields, which showed acoustic noise as the second frequent cause for discomfort with incidence rates of up to 33% of the study population [23,24]. This difference is not as much of a surprise as it appears to be at first glance. Sophisticated UHF brain MR includes anatomical and functional scans with scans times of 10 min and even much longer running at sound pressure levels of up to 112 dB. This setup bears the potential to constitute acoustic noise induced discomfort. In comparison, short breath-hold, cardiac gated scans were used in our UHF-CMR study, which offsets the potential for acoustic noise related discomfort. Another reason for the difference between the rates reported for acoustic noise induced discomfort in our study and previous brain imaging studies might be the use of different acoustic noise protection approaches. In this study earplugs plus headphones were used for acoustic noise protection. Space constraints dictated by the RF coil configurations tailored for brain imaging—where it is common to use a helmet design that closely fits to the head for signal reception—render the use of headphones unsuitable so that acoustic noise protection is primarily accomplished with earplugs [23,24].

The requirements of patient comfort are likely to pave the way for further advances in technology tailored for CMR at 7.0 Tesla, including novel safety concepts [63] and innovative RF coil designs. Though the broad spectrum of applications makes it somewhat challenging to identify a single optimal RF coil design for UHF-CMR, the selected design should meet certain minimum requirements. This should include RF coil casings that afford thermal exchange to offset heat related sensory effects as a root cause for subjective discomfort. These efforts might go as far as printing circuits onto T-shirts or vests that fit the upper torso [64,65]. One could also envision flexible coil designs attached to vacuum pillows, which hold the promise to provide a subject specific fit to the torso while ensuring customized, semi-permanent stabilization. Another important development is the move towards shorter 7.0 T MR systems, which will be far more compatible with installations in clinical imaging suites but which will also help to improve the subjective acceptance during UHF-CMR examinations.

It is a recognized limitation of this study, that only 165 subjects were involved. Taking into account that cardiac MR at 7.0 T is a field in a state of creative flux we felt that it is important to begin by reporting on the details and on the implications of subjective acceptance of UHF-CMR for clinical imaging before the new technology will be placed in the hands of a broader group of clinical colleagues. Also, the study is constrained to subjects undergoing UHF-MR examinations without considering side effects and transient symptoms due to occupational exposure of research and healthcare staff to static or varying magnetic stray fields [66].

## Conclusions

This study adds to the literature by detailing the subjective acceptance of cardiac magnetic resonance imaging examinations at a magnetic field strength of 7.0 T. The most important finding is that all subjects tolerated the UHF-CMR examinations, which is confirmed by no volunteer aborting the examination. Transient muscular contraction and dizziness during the study were the most frequent side effects reported in this study. To conclude, 7.0 T cardiac MR examinations are well tolerated by healthy subjects. Broader observational and multi-center studies including patient cohorts with cardiac diseases together with the use of consistent and simple questionnaires harmonized among UHF-MR institutions [24] are required to provide further insights into the subjective acceptance of UHF-CMR examinations.
